# Impact of Completion of a Pre-Pharmacy Biochemistry Course and Competency Levels in Pre-Pharmacy Courses on Pharmacy Student Performance

**DOI:** 10.3390/pharmacy7030117

**Published:** 2019-08-16

**Authors:** Ruth Vinall, Parto Khansari, Jason McDowell, L. Douglas Ried, Eugene Kreys

**Affiliations:** 1 College of Pharmacy, California Northstate University, Elk Grove, CA 95757, USA; 2 School of Pharmacy and Pharmaceutical Sciences, Stony Brook University Stony Brook, New York, NY 11794, USA; 3 College of Pharmacy, University of New Mexico, Albuquerque, NM 87106, USA

**Keywords:** foundational science courses, pre-pharmacy courses, student performance

## Abstract

Poor performance in foundational science courses, which are usually taken during the first or second year of pharmacy school, can have several negative consequences including increases in student drop-out rates and increases in the number of dismissals and remediating students. The primary goal of the current study was to determine whether completion of a pre-pharmacy biochemistry course and/or performance on a biochemistry competency test (administered at the beginning of the pharmacy program) are associated with pharmacy student performance in foundational science courses and overall academic performance. A secondary goal was to determine whether performance in pre-pharmacy courses and/or student demographics are associated with pharmacy student performance. Prospective univariate analyses (*n* = 75) determined that completion of a pre-pharmacy biochemistry course is not associated with pharmacy student performance. However, performance on a biochemistry competency test was associated with performance in Biochemistry and Cell&Molecular Biology (*p* = 0.002). Furthermore, post-hoc analyses determined that pre-pharmacy cumulative chemistry GPA correlates with performance in both the Biochemistry and Cell&Molecular Biology and Medicinal Chemistry foundational science courses (*p* = 0.002 and *p* = 0.04, respectively) and can predict first year GPA (*p* = 0.002). The combined data indicate that further assessment of the impact of pre-pharmacy competency in biochemistry and chemistry on pharmacy student success is warranted.

## 1. Introduction

The highest levels of pharmacy student failure and attrition are typically observed during the first and second years of pharmacy school when foundational science courses such as biochemistry and medicinal chemistry are usually taught [1,2]. Student failure has a large negative impact on both pharmacy programs and students. For pharmacy programs, loss of students can jeopardize program accreditation while increases in the number of remediating students can strain program resources. It is noteworthy that many students who fail foundational science courses continue to underperform throughout their pharmacy program; course failure in the first year correlates strongly with course failure in subsequent academic years and with board exam failure [1,3,4,5,6]. For students, course failure can cause significant financial loss as well as increased stress levels.

Multiple research studies have been conducted to help identify predictors of students’ success and to help guide the development of strategies which can improve student oucomes. Several of these have demonstrated that overall grade point average (GPA), science and math GPA, and age can predict student success in some settings [2,3,4,7,8,9,10], and many colleges use some or all of these metrics as part of their admissions criteria. Pharmacy programs also require completion of selected pre-pharmacy courses to help ensure that students are adequately prepared for the rigorous pharmacy curriculum. There is much debate as to which pre-pharmacy courses are essential for student success, and, as might be expected, review of pre-pharmacy courses required for admission to pharmacy programs throughout Northern America, as well as throughout the world, shows that these vary widely [11,12,13,14,15]. The vast majority of pharmacy programs require completion of general and organic chemistry courses as well as a course in general biology prior to admission, however, the requirement for biochemistry and other higher level chemistry and biological science courses is more varied. For example, according to a 2009 study of 71 colleges of pharmacy in Northern America, only 14 of them required biochemistry as a pre-pharmacy course, i.e., only 19.7% [11]. It is noteworthy that schools which offer integrated pre-pharmacy/pharmacy programs often include biochemistry as part of their pre-pharmacy curriculum. Studies have demonstrated that successful completion of a pre-pharmacy biochemistry course is associated with higher cumulative first and second year GPA [4,16], however, these studies did not determine the impact completing a pre-pharmacy biochemistry course on performance in individual foundational science courses, and did not assess competency in biochemistry at the beginning of the pharmacy program. It is also noteworthy that, to our knowledge, the impact of performance in individual pre-pharmacy subject areas on pharmacy student success, with the exception of math, have not been assessed. A challenge here is that while overall GPA as well as science and math GPA are student metrics which are typically available via national pharmacy application services such as PharmCAS, performance in other subject areas must be captured and calculated by individual institutions.

In the current study, we assess the impact of completion of a pre-pharmacy biochemistry course as well as performance on a baseline biochemistry competence test (administered at the beginning of the pharmacy program) on student performance in foundational science courses (Biochemistry and Cell&Molecular Biology and Medicinal Chemistry) and first year GPA. We also perform an exploratory analysis to determine whether performance in specific categories of pre-pharmacy courses is associated with student success.

## 2. Materials and Methods

### 2.1. Data Collection

This study was approved by the California Northstate University College of Pharmacy (CNUCOP) Institutional Review Board (IRB) for the Protection of Human Subjects and data was only collected from the students who signed consent forms. Of the 115 students enrolled in first year pharmacy program, 75 students participated in this study (65.8% response rate). Student demographic data such as age and gender, pre-pharmacy course data, and the time between when the pre-pharmacy course was taken relative to when they entered the PharmD program, was primarily obtained through PharmCAS. When data was not available through PharmCAS (e.g., because the student had completed a course after conditional acceptance into the CNUCOP PharmD program), students’ supplemental applications and/or student transcripts were utilized to obtain these data. In all cases cumulative GPA (e.g., cumulative chemistry GPA) was calculated by assessing both scores and semester units, i.e., the same method used by PharmCAS to assess individual subject performance. Student performance data for the PharmD foundational science courses were obtained from course coordinators; student GPA was provided by the Office of the Registrar.

### 2.2. Study Variables

Predictor variables included successful completion of a pre-pharmacy biochemistry course, course grades for all pre-pharmacy courses required for admission to CNUCOP, pre-pharmacy science GPA, pre-pharmacy GPA, and demographic data. Note that successful completion of the pre-pharmacy biochemistry course was defined as the student having scored grade ‘C’ or above. These data were collected from the admissions records of consented students. Performance on biochemistry tests were also used to predict student outcomes. These were administered prior to and after the delivery of the biochemistry block of the Biochemistry and Cell&Molecular Biology course (these tests are referred to as the biochemistry pre-test and biochemistry post-test). The biochemistry pre-test comprised of 10 multiple choice questions. The post-test comprised of 20 multiple choice questions which included the ten original questions from the pre-class test and an additional ten questions to limit the potential for recalling the answers to the question rather than understanding the concept [17]. Note that each multiple choice question had 5 answer options, and that paper tests were administered and graded by the instructor. Negative marking was not employed. The Biochemistry and Cell&Molecular Biology courses and the Medicinal Chemistry course are both mandatory for CNUCOP PharmD students and are offered during the fall semester of the first year.

Outcome variables which were assessed as part of this study included: (1). Performance on the Biochemistry and Cell&Molecular Biology course final exam, (2). Performance in the Medicinal Chemistry course, (3). Cumulative first year fall semester GPA, and (4). Cumulative first year GPA. 

### 2.3. Statistical Analyses

Means and standard deviations were calculated for interval level data and percentages were calculated for nominal and ordinal data. The bivariate tests of mean differences in the academic outcomes were conducted using t-tests for variables with two categories and ANOVA for non-numeric variables with more than two categories. The associations among numeric variables were analyzed using Pearson’s correlation. The joint influence of the preadmission academic performance on the prediction of first academic year performance was evaluated using multivariable ordinary least squares regression. The independent effect of the pharmacy pre-requisites upon GPA was evaluated using hierarchical multiple regression techniques. Multivariable analysis was performed to adjust for incoming cumulative GPA and certain demographic factors such age and gender. If the change in explained variance (r^2^) was statistically significant, then addition of the biochemistry variable added significantly to the prediction of fall and cumulative GPA. IBM SPSS Statistics Version 20 was used to conduct the statistical analyses (IBM Corp. Released 2011. IBM SPSS Statistics for Windows, Version 20.0. Armonk, NY: IBM Corp). The *a priori* level of statistical significance was alpha = 0.05 (2-sided).

## 3. Results

### Descriptive Statistics

Seventy-five students in the CNUCOP class of 2017 cohort (65.8%) signed the informed consent to allow measures of their academic performance and demographic data to be used in this study. Among the 75 students participating in the study, 61 students (81.3%) had completed a pre-pharmacy biochemistry course and 14 students (18.7%) had not. No significant differences were observed between these two groups in regards to overall and science GPA and student demographics (Table 1).

Completion of a pre-pharmacy course in biochemistry did not impact student performance in the Biochemistry and Cell&Molecular Biology and Medicinal Chemistry first year foundational sciences courses.

A statistically significant difference in performance in the pharmacy program Biochemistry and Cell&Molecular Biology course (final course grade, and the Biochemistry section block exam) and the Medicinal Chemistry course (final course grade) was not observed between pharmacy students who had successfully completed a pre-pharmacy course in biochemistry versus those who had not (Table 2). While the mean scores were lower for students in the ‘no pre-pharmacy biochemistry course’ group, statistical significance was not achieved. The combined data indicate that completion of a pre-pharmacy course in biochemistry does not result in improved performance in a pharmacy program foundational science courses.

Pre-course competency in biochemistry (assessed by a pre-test) is associated with performance in the Biochemistry and Cell&Molecular Biology course. 

Pearson correlation coefficient analyses determined that associations exist between performance on the biochemistry pre-test (administered at the beginning of the pharmacy program prior to the start of the Biochemistry and Cell&Molecular Biology course) and performance in the Biochemistry and Cell&Molecular Biology course (Table 3; *p* = 0.002). A strong correlation existed between performance on the biochemistry pre- and post-test was observed (Table 3 and Table 4; *p* = 0.001), and correlations between post-test performance and performance in the Biochemistry and Cell&Molecular Biology course (Table 3; *p* = 0.019) as well as the Medicinal Chemistry course (Table 4; *p* = 0.001) were observed. The combined data indicate that assessment of biochemistry knowledge at the beginning of a pharmacy program is a better indicator of performance in pharmacy program foundational science courses than assessment of completion of a pre-pharmacy biochemistry course. It is noteworthy that there was no difference in pre-test performance in students who had versus had not taken a pre-pharmacy biochemistry course (*p* = 0.30). We hypothesized that this may be due in part to the amount of time between when students took the pre-pharmacy biochemistry course. Subsequent analysis of the impact of the amount of time between when the undergraduate biochemistry course was taken and when students took the biochemistry pre-test did not correlate with student performance on the pretest (r^2^ = 0.01, *p* = 0.54), indicating that knowledge retention is not likely responsible for our observation. Other confounding factors which may help explain the discrepancy are type of school where the pre-pharmacy course was taken and overall number of pre-pharmacy science courses taken by students. Our finding supports the inclusion of a biochemistry competency test at the beginning of a pharmacy program. The information from this test could be used to support and guide the implementation of supplemental instruction and tutoring in biochemistry for students who perform poorly on the test.

An association between performance on the biochemistry pre-test and performance in the Medicinal Chemistry course was not observed, however, an association between the biochemistry post-test and performance in the Medicinal Chemistry course was observed (*p* = 0.001) (Table 4). It is noteworthy that there was a strong correlation between student performance in the Biochemistry and Cell&Molecular Biology course and performance in the Medicinal Chemistry course (r^2^ = 0.61, *p* < 0.001). The combined data indicate that improving student performance in Biochemistry could help improve performance in both the Biochemistry and Cell&Molecular Biology and Medicinal Chemistry foundational science courses.

Completion of a pre-pharmacy biochemistry course and pre-course biochemistry knowledge is not associated with first year cumulative GPA.

A secondary hypothesis of the study was that completion of a pre-pharmacy biochemistry course and/or pre-course biochemistry knowledge would not impact pharmacy student first year cumulative GPA. Our data support this hypothesis; completion of a pre-pharmacy biochemistry course did not have a statistically significant impact on first year fall semester GPA (Table 2; 3.18 versus 3.00, unadjusted *p* = 0.13) or first year cumulative GPA (Table 2; 3.28 versus 3.20, unadjusted *p* = 0.54).

Association of student performance in other pre-pharmacy courses with student performance in the Biochemistry & Cell and Molecular Biology course and Medicinal Chemistry course, and first year cumulative GPA. 

Our exploratory analyses determined that there are several associations between performance in pre-pharmacy science courses and student success in the Biochemistry and Cell&Molecular Biology course and the Medicinal Chemistry course. Students’ final course grade in the Biochemistry and Cell&Molecular Biology course was associated with students’ pre-pharmacy academic performance, namely pre-pharmacy science GPA (r^2^ = 0.18, *p* < 0.001), pre-pharmacy GPA (r^2^ = 0.37, *p* < 0.001), and cumulative pre-pharmacy chemistry GPA (r^2^ = 0.06, *p* = 0.04) (Table S1). Students’ final course grade in the Medicinal Chemistry course was associated with pre-pharmacy science GPA (r^2^ = 0.20, *p* < 0.001), pre-pharmacy GPA (r^2^ = 0.14, *p* < 0.001), cumulative pre-pharmacy chemistry GPA (r^2^ = 013, *p* = 0.002) as well as age (r^2^ =0.13, *p* = 0.01) (Table S2).

Similar associations were observed between these predictor variables and first year pharmacy cumulative GPA; first year pharmacy student GPA was associated with pre-pharmacy science GPA (r^2^ = 0.17, *p* < 0.001), pre-pharmacy cumulative GPA (r^2^ = 0.19, *p* < 0.001), age (r^2^ = 0.10, *p* = 0.008), and cumulative chemistry GPA (r^2^ = 0.12, *p* = 0.002) (Table S3). Other than cumulative chemistry GPA, no other associations were observed between first year pharmacy student GPA and performance in pre-pharmacy courses (general biology, microbiology, physiology, anatomy, physics, calculus, statistics, economics, psychology, public speaking, biochemistry/cell&molecular biology (Appendix A Table A1).

## 4. Discussion

To our knowledge, the impact of completion of pre-pharmacy biochemistry and student performance in pre-pharmacy courses on subsequent performance in pharmacy program foundational science courses has not previously been investigated directly. In the current study, we demonstrate that completion of pre-pharmacy biochemistry does not impact performance in the Biochemistry and Cell&Molecular Biology or the Medicinal Chemistry foundational sciences courses, however, we show that competency in biochemistry at the beginning of a pharmacy program (assessed using a pre-test) is strongly associated with subsequent performance in the Biochemistry and Cell&Molecular Biology course, and that performance in the Biochemistry and Cell&Molecular Biology and Medicinal Chemistry courses is tightly linked. The combined data support current American Association of Colleges of Pharmacy (AACP) guidelines which indicate completion of a pre-pharmacy biochemistry course need not be required as a prerequisite for entry into a pharmacy program [11].

It is noteworthy that an association between time since taking a pre-pharmacy biochemistry course and performance on the pre-test was not observed, and that students who had versus had not taken a pre-pharmacy biochemistry course performed similarly on the pre-test. This, combined with the fact that performance in the pre-test and subsequent performance in the Biochemistry and Cell&Molecular Biology course, indicate that using assessment of whether or not a student has taken pre-pharmacy biochemistry is not helpful in the identification of students who may benefit from supplemental instruction, and, instead, support continued implementation of a pre-test to help identify ‘at risk’ students. This strategy could help improve student performance in pharmacy program foundational science courses and thereby help abrogate the negative outcomes which are associated with failure and underperformance in foundational science courses [1,2,3,4,5,6]. Studies have demonstrated that providing tutoring and supplemental instruction to pharmacy students can improve outcomes [18,19].

McCall et al. have previously reported that successful completion of a pre-pharmacy biochemistry course is correlated with a higher cumulative first year GPA at Texas Tech College of Pharmacy [4]. We did not observe this association, however, it is difficult to compare the two studies due to significant differences between the two institutions admissions policies; admission into CNUCOP at the time of this study required completion of a 4 year undergraduate degree while Texas Tech College of Pharmacy accepted students with a 2 year undergraduate degree (only 34% of Texas Tech College of Pharmacy students enrolled in the study had completed a 4 year degree). McCall et al. did not look at the impact of the pre-pharmacy biochemistry performance on individual course performance during the first year of pharmacy school, however, the study did show that pharmacy students with a bachelor of science (BS) degree and who had completed advanced biology course work were more likely to graduate from the pharmacy program without academic delay or suspension compared to students with a 2 year degree, while students with a bachelor of art (BA) degree had rates of academic delay and suspension which were similar to students with a 2 year degree [4]. These data further underscore the importance of foundational science subject knowledge for student success in pharmacy programs. 

Our exploratory analyses identified cumulative chemistry GPA, overall GPA, pre-pharmacy science GPA, and age, as predictors of student performance in both the pharmacy program Medicinal Chemistry and the Biochemistry and Cell&Molecular Biology foundational science courses. These measures also correlated with first year cumulative GPA. Multiple other studies have found a correlation between pre-pharmacy overall and science GPA with pharmacy student cumulative first year GPA [2,3,4,7,8]. McCall et al. also demonstrated a correlation with age, considered by many a surrogate measure for maturity, and showed it is a predictor of academic success [20]. While other studies have demonstrated that cumulative first year GPA is associated with pre-pharmacy organic chemistry and PCAT chemistry scores [3,21], to our knowledge, our study is the first study to identify cumulative chemistry GPA as a predictor of foundational science course performance and pharmacy student cumulative first year GPA. It is noteworthy that pre-pharmacy cumulative chemistry GPA is not available through PharmCAS and in this study was calculated through analysis of supplemental applications, hence usage of this variable to predict performance may be impractical for some colleges.

The major limitations of this study were sample size (*n* = 75) and its restriction to a single college of pharmacy. Larger and ideally multi-school studies will be needed to validate our findings and to broaden their utility. It should also be noted that students who agreed to participate in the study generally scored higher in the Biochemistry & Cell and Molecular Biology course (85.4% versus 80.5%, *p* = 0.001) and had a higher GPA (3.14 versus 2.77, *t* = 3.70. *p* = 0.001) compared to those who did not agree to participate. These findings indicate internal validity bias may exist for this study and the data should be interpreted with caution.

In summary, our combined data provide evidence that support current AACP guidelines which state completion of pre-pharmacy biochemistry is not necessary for admission into a pharmacy program. They also support the implementation of a biochemistry competency test at the beginning of a pharmacy program to help identify students who would benefit from supplemental instruction and tutoring. Lastly, our data indicate that validation of cumulative chemistry GPA as a predictor of academic success in a pharmacy program is warranted.

## Figures and Tables

**Table 1:** Student characteristics of consented students who did versus did not successfully complete a pre-pharmacy biochemistry course.

	Successful Completion of a Pre-Pharmacy Biochemistry Course		
	Yes (*n* = 61)	No (*n* = 14)	*p* Value
Academic characteristics;			
Science GPA (mean)	2.93	2.90	0.77
Overall GPA (mean)	3.11	3.06	0.61
Age (mean)	26.2	27.6	0.29
Female Gender (%)	63.9	57.1	0.64
Race (%)			
Asian	63.9	71.4	0.67
White	31.1	28.6	
Hispanic	4.9	0.0	

**Table 2:** Comparison of the impact of successful completion of an undergraduate biochemistry course on student performance in PharmD foundational science courses and first year GPA.

			Unadjusted Analysis		Adjusted Analysis	
	Biochemistry Pre-Requisite (Mean Score, %)	No Biochemistry Pre-Requisite (Mean Score, %)	Mean Difference (95% CI)	*p* Value	Mean Difference (95% CI)	*p* Value
* Biochem and C&MB, final course grade (mean% ± SD)	85.73 ± 6.84	84.08 ± 7.60	1.65 (−2.48–5.77)	0.43	1.47 (−2.36–5.30)	0.45
Biochem block exam for the *Biochem and C&MB course (mean% ± SD)	86.08 ± 9.24	85.46 ± 10.93	0.62 (−5.03–6.27)	0.83	0.73 (−4.72–6.17)	0.79
Medicinal chemistry, final course grade (mean% ± SD)	82.63 ± 6.86	79.69 ± 10.24	2.9 (−1.53–7.41)	0.19	3.06 (−1.00–7.11)	0.14
P1 Fall semester GPA ± SD	3.18 ± 0.41	3.00 ± 0.62	0.2 (−0.6–0.47)	0.13	0.22 (−0.03–0.47)	0.08
P1 GPA ± SD	3.28 ± 0.39	3.20 ± 0.48	0.08 (−0.18–0.33)	0.54	0.11 (−0.13–0.35)	0.36

* Biochem and C&MB = Biochemistry and Cell&Molecular Biology.

**Table 3:** Associations between biochemistry pre-test and post-test scores * and performance in Biochemistry and Cell&Molecular Biology (Biochem and C&MB).

		Biochem and C&MB Course Score (%)	Biochemistry Pre-Test (%)	Biochemistry Post-Test (%)
Biochem and C&MB course score (%)	R^2^ (*p*-value)	1	0.359 (0.002)	0.270 (0.019)
Biochemistry pre-test (%) *	R^2^ (*p*-value)	0.359 (0.002)	1	0.519 (0.001)
Biochemistry post-test (%) *	R^2^ (*p*-value)	0.270 (0.019)	0.519 (0.001)	1

* Pre-test was administered at the beginning of the pharmacy program, the post-test was administered following completion of the biochemistry block of the Biochemistry and Cell&Molecular Biology course.

**Table 4:** Associations between biochemistry pre-test and post-test scores * and performance in Medicinal Chemistry.

		Medicinal Chemistry Course Score (%)	Biochemistry Pre-Test (%)	Biochemistry Post-Test (%)
Medicinal Chemistry course score (%)	R^2^ (*p*-value)	1	0.168 (0.149)	0.395 (0.001)
Biochemistry pre-test (%) *	R^2^ (*p*-value)	0.168 (0.149)	1	0.519 (0.001)
Biochemistry post-test (%) *	R^2^ (*p*-value)	0.395 (0.001)	0.519 (0.001)	1

* Pre-test was administered at the beginning of the pharmacy program; the post-test was administered following completion of the biochemistry block of the Biochemistry and Cell&Molecular Biology course.

## Supplementary Materials

The following are available online at https://www.mdpi.com/2226-4787/7/3/117/s1.

## Appendix A

**Table A1:** Academic admission requirements for California Northstate University College of Pharmacy (CNUCOP) at the time of this study (2013).

Required Pre-Pharmacy Courses	Semester Hours	Quarter Hours
General Chemistry *	8	12
Organic Chemistry *	8	12
Biochemistry, or Cell & Molecular Biology	3	4
General Biology *	8	12
Microbiology	3	4
Physiology ***	4	6
Anatomy ***	4	6
Physics **	4	6
Calculus **	3	4
Statistics	3	4
Economics (Micro or Macro)	3	4
Psychology	3	4
Public Speaking	3	4
	TOTAL = 57 h	TOTAL = 82 h

* Course must have lab component or lab equivalent, ** AP is acceptable, *** 1 year of combined Anatomy & Physiology is acceptable.
