# Supplementary material for: Impact of Completion of a Pre-Pharmacy Biochemistry Course and Competency Levels in Pre-Pharmacy Courses on Pharmacy Student Performance
Source: Pharmacy (Basel). 2019 Aug 16;7(3):117. doi: 10.3390/pharmacy7030117 (PMC6789439; doi:10.3390/pharmacy7030117)
Supplement: Supplementary file 1 [file pharmacy-07-00117-s001.pdf]

Table 1. Regression analysis determined pre-pharmacy GPA and science GPA, and cumulative pre-pharmacy chemistry GPA can predict student performance in the Biochemistry & Cell and Molecular Biology course.

Regression on Course Percentage							
Variable Name	Pearson r (2-tailed)	B-Coeff (95% CI)	Std Coeff (Beta)	t-test Sig	B-Coeff (95% CI)	Std Coeff (Beta)	t-test Sig
Prepharmacy Science GPA	0.43 P<0.001	9.10 0.12-18.09	0.47	2.02 0.05	9.18 0.17-18.19	0.47	2.03 .05
Prepharmacy GPA	0.37 p-0.001	-0.37 -11.13-10.38	-0.02	-0.07 0.95	-0.60 -11.38-10.19	-0.02	-0.11 0.91
Age	0.18 P=0.12	0.06 -0.32-0.44	0.04	0.32 0.75	0.10 -0.29-0.49	0.07	0.53 0.60
Cumulative Prepharmacy Chemistry GPA	0.24 P=0.04	-1.13 -5.43-3.17	-0.08	-0.52 0.60	-1.33 -5.66-3.00	-0.09	-0.61 0.54

Table S2: Regression analysis determined pre-pharmacy GPA and science GPA, student age, and cumulative pre-pharmacy chemistry GPA can predict student performance in the Medicinal Chemistry course.

**Regression on Medicinal Chemistry Class Percentage**							
Variable Name	Pearson r (2-tailed)	B-Coeff (95% CI)	Std Coeff (Beta)	t-test Sig	B-Coeff (95% CI)	Std Coeff (Beta)	t-test Sig
Prepharmacy Science GPA	0.45 P<0.001	7.74 -1.78-17.28	0.37	1.62 0.11	7.86 -1.62-17.35	0.38	1.66 0.10
Prepharmacy GPA	0.38 P=0.001	-1.37 -12.77-10.03	-0.05	-0.24 0.81	-1.72 -13.07-9.64	-0.06	-0.30 0.76
Age	0.28 P=0.01	0.18 -0.22-0.57	0.11	0.88 0.38	0.24 -0.17-0.65	0.15	1.18 0.24
Cumulative Prepharmacy Chemistry GPA	0.36 P=0.002	1.49 -3.07-6.05	0.10	0.65 0.52	1.17 -3.39-5.74	0.08	0.51 0.61

Table S3: Regression analysis determined pre-pharmacy GPA and science GPA, student age, and cumulative pre-pharmacy chemistry GPA can first year GPA.

**Regression on Semester GPA**							
Variable Name	Pearson r (2-tailed)	B-Coeff (95% CI)	Std Coeff (Beta)	t-test Sig	B-Coeff (95% CI)	Std Coeff (Beta)	t-test Sig
Prepharmacy Science GPA	0.41 P<0.001	0.04 -0.53-0.61	0.03	0.14 0.89	0.05 -0.52-0.62	0.04	0.18 0.86
Prepharmacy GPA	0.44 P=0.001	0.47 -0.22-1.16	0.28	1.36 0.17	0.45 -0.23-1.13	0.27	1.31 0.20
Age	0.31 P=0.008	0.01 -0.01-0.04	0.15	1.19 0.24	0.02 -0.01-0.04	0.19	1.55 0.13
Cumulative Prepharmacy Chemistry GPA	0.35 P=0.002	0.10 -0.17-0.38	0.11	0.74 0.46	0.08 -0.19-0.35	0.09	0.58 0.56
